# A Signature of Senescence Based on An Analysis of Selected Markers in Primary Vascular Smooth Muscle Cells Cultured *In Vitro* and Derived from Atherosclerotic Plaque

**DOI:** 10.14336/AD.2025.0687

**Published:** 2026-10-12

**Authors:** Agata Głuchowska, Krzysztof Bojakowski, Dorota Janiszewska, Karolina Staniak, Magdalena Dudkowska, Grażyna Mosieniak

**Affiliations:** ^1^Laboratory of Molecular Bases of Aging, Nencki Institute of Experimental Biology, Polish Academy of Sciences, Warsaw, Poland.; ^2^Laboratory of Cytometry, Nencki Institute of Experimental Biology, Polish Academy of Sciences, Warsaw, Poland.; ^3^Department of Plant Genetics, Breeding and Biotechnology, Institute of Biology, Warsaw University of Life Sciences, Warsaw, Poland.; ^4^Clinical Department of General and Vascular Surgery, National Medical Institute of the Ministry of the Interior and Administration, Warsaw, Poland.; ^5^2nd Department of Vascular Surgery and Angiology, Centre of Postgraduate Medical Education, Warsaw, Poland.; ^6^Laboratory of Calcium Binding Proteins, Nencki Institute of Experimental Biology, Polish Academy of Sciences, Warsaw, Poland.; ^7^Laboratory of Biomolecular Interactions Studies, Chair of Drug and Cosmetics Biotechnology, Faculty of Chemistry, Warsaw University of Technology, Warsaw, Poland

**Keywords:** senescence, vascular smooth muscle cells, biomarkers, p16

## Abstract

Cellular senescence is a state of stable growth arrest that is accompanied by a characteristic phenotype. The senescent phenotype is dynamic and heterogeneous, and it depends on both the type of cell and the factors that trigger senescence. There is no single biomarker that is shared by all senescent cell types. Therefore, our studies aimed to define a set of biomarkers that can discriminate among proliferating, senescent and temporally growth-arrested (quiescent) cells. We analysed vascular smooth muscle cells (VSMCs) derived from healthy aortae that underwent senescence *in vitro* but also VSMCs isolated from human atherosclerotic plaques that underwent senescence *in vivo*. Our results demonstrate that changes in the levels of nuclear proteins (Lamin B1, HMGB1, PARP1) are the most consistent, stable and widespread in cells senescing *in vitro* while changes in the levels of signalling proteins implicated in senescence (p53, p21^CIP^, p16^INK4A^) appeared to be more variable. VSMCs derived from atherosclerotic plaques reflects pattern of senescence biomarker expression observed in VSMCs undergoing *in vitro* senescence but some differences were revealed. There was a gradual decrease in p21^CIP^ level and an increase in p16^INK4A^ level as VSMCs cultured *in vitro* progressed toward late senescence or entered replicative senescence, whereas plaque-derived senescent VSMCs showed increased p21^CIP^ and p16^INK4A^ protein expression compared with proliferating VSMCs from healthy aortas. Overall, we have defined a set of biomarkers that is common for VSMCs undergoing *in vitro* and *in vivo* senescence and enable to recognize senescent cells with high confidence, regardless of senescence trigger and senescence phase (early versus late).

## INTRODUCTION

Aging is characterized by a progressive loss of physiological integrity, leading to impaired function and increased vulnerability to death [1]. This deterioration is the primary risk factor for major human pathologies including cardiovascular disorders such as atherosclerosis, which represents one of the main causes of death in developed countries. Number of studies have demonstrated that cellular senescence is an important contributor to aging and age-related diseases [2]. Senescent cells accumulate with age and an increased number of such cells were found at sites of age-related pathologies like atherosclerosis [3]. Atherosclerosis is an inflammatory disease of the arteries characterized by atherosclerotic plaque formation due to the accumulation of lipids, inflammatory cells, apoptotic cells, calcium and extracellular matrix proteins [4]. The most dangerous stage of plaque development is the formation of an unstable plaque that is prone to rupture. This phenomenon can lead to thrombus formation, vessel occlusion, and ultimately, stroke or myocardial infraction. Importantly, senescent vascular smooth muscle cells (VSMCs) have been identified in atherosclerotic plaques. Furthermore, it has been postulated that senescent VSMCs present in the fibrous cap of atherosclerotic plaques may significantly contribute to ineffective plaque repair, causing plaque instability and rupture [5, 6].

Cellular senescence is defined as a state of persistent cell cycle arrest into which cells enter in response to various stressors, such as DNA damage, oxidative stress, or oncogene activation [7]. This process was first observed by Leonard Hayflick in 1965, who noted that primary human fibroblasts can only divide a finite number of times before entering a nondividing state [8]. Although senescent cells lose their ability to proliferate, they remain metabolically active and secrete a variety of bioactive molecules, collectively known as the senescence-associated secretory phenotype (SASP). Unlike quiescent cells, which are dormant but retain the ability to re-enter the cell cycle and proliferate in response to appropriate growth signals or stimuli, senescent cells are insensitive to growth factors and mitogenic stimuli that typically initiate cell division.

Cellular senescence is a multistep process during which a series of changes occur in the cell, leading to the acquisition of a specific phenotype [9-11]. Cellular senescence is usually initiated by the induction of DNA damage and the activation of the DNA damage response (DDR) pathway. As a consequence, the upregulation of cell cycle-regulating proteins, such as p53, p16INK4a (p16), p21CIP1 (p21), and p-Rb, is observed. Importantly, DDR activation is a long-lasting event that accompanies stable cell cycle arrest in senescent cells. Moreover, increased activity of the lysosomal enzyme β-galactosidase detected at pH 6.0, called senescence-associated β-galactosidase (SA-β-gal), is a common and fundamental marker of senescent cells. During senescence, cells undergo a massive reorganization of morphology and intracellular structure, leading to changes in nuclear architecture and composition, such as the loss of Lamin B1, which is a nuclear envelope protein, the formation of senescence-associated heterochromatin (SAHF) foci, mitochondrial dysfunction, lysosomal accumulation, and other changes. The characteristic feature of senescent cells that has the greatest functional importance is their diverse senescence-associated secretory phenotype (SASP), which affects neighbouring cells and influences the tissue microenvironment.

Advances in knowledge and technology have allowed scientists to eliminate senescent cells from laboratory animals, which extends the life of these animals and prevents or delays the development of age-related diseases, including atherosclerosis [2, 12, 13]. However, despite progress in understanding the role of cellular senescence in the ageing of organisms, providing a clear definition and description of the cellular senescence phenotype remains difficult [14]. The influx of new data over the last decade has resulted in the establishment of a set of features referred to as senescence markers [10, 15]. Scientists agree that none of these markers are unique, specific or sufficient to identify senescent cells, especially since this process appears to be highly dynamic, and the properties of senescent cells change over time and may depend, for example, on the type of tissue from which they originate [15-17]. Instead, a composition of molecular factors that appear simultaneously or subsequently is observed. Thus, the identification and characterization of senescent cells, particularly those undergoing senescence in vivo, are still under research [18].

The aim of our study therefore was to define a set of selected markers that would most accurately correlate with the senescent phenotype of VSMCs. To this end, we analysed cells that were induced to undergo senescence by different triggers (doxorubicin or H_2_O_2_) and compared senescent cells with proliferating cells and temporarily growth-arrested, quiescent cells. Additionally, we include in our studies other cell types, namely fibroblasts and preadipocytes. We verified the usefulness of selected markers to reveal *in vivo* senescence of VSMCs derived from atherosclerotic plaques. Based on a limited but highly analysable set of features that are commonly considered to be characteristic of senescent cells, the results revealed differences and similarities in senescent phenotypes, which may be useful for better assessing this process particularly in VSMCs.

## MATERIALS AND METHODS

### Cell lines

Primary human vascular smooth muscle cells (VSMCs) were purchased from ATCC and cultured in vascular cell basal medium (ATCC, LGC, Poland) supplemented as defined by the manufacturer. Human primary dermal fibroblasts were purchased from ATCC and cultured in DMEM medium (Sigma-Aldrich) supplemented with 10% fetal bovine serum (Biowest), 2mM L-glutamine and Antibiotics Antimycotic solution (Sigma-Aldrich). Human primary preadipocytes were purchased from ScienCell and cultured in dedicated medium (ScienCell) supplemented as defined by the manufacturer.

### Isolation of smooth muscle cells from atherosclerotic plaques.

Smooth muscle cells were isolated from atherosclerotic plaques obtained from patients who underwent carotid endarterectomy. Studies involving human samples were approved by the Ethics Committee of the Central Clinical Hospital of the Ministry of Internal Affairs. Written informed consent was obtained from all patients. Fresh isolated atherosclerotic plaques were collected in DMEM medium supplemented with 10% fetal bovine serum, 2mM L-glutamine, 10 U/ml penicillin, 0.1 mg/ml streptomycin and 0.25 ug/ml amphotericin B, on ice within 30 min of operative resection. Plaques fragments were then dissected into 2-3 mm^2^ pieces and ten to fifteen pieces were placed in each well of a six-well plate, pre-coated with collagen (Becton Dickinson) and cultured in DMEM medium in a humidified atmosphere (37°C and 5% CO2 in the air). Tissue explants were kept under cell culture conditions for up to 4 weeks to enable cell migration from explants. Cells derived from atherosclerotic plaque were trypsinized with 0.25% trypsin solution (Sigma-Aldrich) and collected to prepare protein extracts or seeded at a density of approximately 3500 cells/cm^2^ in a medium dedicated for vascular smooth muscle cells and subjected to other analysis within 1-2 days. Cells from each plaque have been analyzed separately.

### Senescence induction

To obtain population of cells that underwent stress-induced premature senescence (SIPS), VSMCs, preadipocytes and fibroblasts were seeded at a density of 3,500 cells/cm^2^. Twenty- four hours after seeding cells were treated with doxorubicin (150 nM) or H_2_O_2_ (150 μM) and cultured for 3 days or 7 days without changing the medium. In some experiments cells treated with H_2_O_2_ (150 μM) were cultured for 4 weeks and medium was changed once a week.

To obtain a population of cells that underwent replicative senescence, VSMCs were passaged every 3-4 days till they lost proliferative potential.

### Induction of quiescence

Induction of quiescence was performed according to Mitra at al. [19]. Briefly, to induce quiescence by contact inhibition (Qi) intensively proliferating VSMCs, preadipocytes and fibroblasts were seeded at a density of 3500 cells/cm^2^ and cultured for next 8 days with changing medium every 48 hours. To induce quiescence by serum starvation (Qs) cells were seeded at a density of 3,500 cells/cm^2^. After 24 hours, cells were washed with PBS and then cultured in a medium without serum, containing only dedicated supplements (VSMC and preadipocytes) or in a low serum medium DMEM with 0.5% FBS (fibroblasts) for 7 days with changing medium every 48 hours.

### Detection of senescence-associated β-galactosidase

Detection of senescence-associated β-galactosidase (SA-β-gal) was performed according to Dimri et al. [20]as previously described [21]. Briefly, cells were fixed in 2% formaldehyde, 0.2% glutaraldehyde in PBS, washed, and incubated overnight at 37 °C with a solution containing 1 mg/ml 5-bromo-4-chloro-3-indolyl-β-d-galacto-pyranoside, 2.5 mM potassium ferrocyanide, 2.5 mM potassium ferricyanide, 150 mM NaCl, 2 mM MgCl_2_ and 0.1 M phosphate buffer, pH 6. Cells nuclei were stained with DAPI (1 μg/mL in PBS) (Sigma-Aldrich, D9542). Cells were observed under a Nikon Eclipse microscope with a 40×/ 0.75 Nikon lens and the ImagePro Plus.

### BrdU incorporation assay

To investigate DNA synthesis, 10 μM of 5-bromo-2’-deoxyuridine (BrdU; Sigma-Aldrich) was added to the medium and cells were cultured for 18 h. BrdU-positive cells were detected using a primary mouse antibody against BrdU (10μM; Becton-Dickinson) and anti-mouse Alexa 488 (1:500) (Invitrogen) secondary antibody. To visualize all nuclei, cells were stained with DAPI (1 μg/mL; Sigma-Aldrich). Cells were observed under a fluorescence microscope (Nikon), photographed using a DS-Fi2 camera with a 40x/0.75 Nikon lens and counted using ImageJ software.

### Immunocytochemical detection of Ki67

Cells were fixed with ice-cold 70% ethanol and stored at -20°C for at least 24 h. Samples were blocked for 30 min with 5% BSA in PBS containing 0.5% Tween and 0.1% Triton X-100 (Sigma-Aldrich), washed, and incubated for 2 h with a primary antibody against Ki67 (Abcam). After washing with PBS, cells were incubated for 1 h with a secondary anti-rabbit antibody conjugated with Alexa 488 and for 15 min with DAPI (1 μg/mL) to stain all nuclei. Cells were observed under a fluorescence microscope (Nikon) and photographed using a DS-Fi2 camera (Nikon). At least 40 cells were analyzed for each condition (proliferating, quiescent and senescent cells) in each experiment.

### Cell cycle analysis

For cell cycle analysis, the cells were fixed in 70% ethanol and stained with PI solution (3.8 mM sodium citrate, 500 μg/mL RNAse A and 50 μg/mL PI in PBS) (Sigma-Aldrich). DNA content was assessed using flow cytometry (FACSCalibur with Modfit Software—Becton Dickinson, Franklin Lakes, NJ, USA). Ten thousand events were collected per sample.

### Western blotting

Whole cells protein extracts were prepared according to the Laemmli method [22]. Equal amounts of protein were separated electrophoretically in 10, 12 or 15% SDS-polyacrylamide gels and transferred to nitrocellulose membranes. Membranes were blocked with 5% non-fat dry milk dissolved in TBS containing 0.1% Tween 20 for 1h at RT and then incubated overnight at 4°C with one of the primary antibodies: rabbit anti-p21 (1:500, Santa Cruz), rabbit anti-p16 (1:500, Proteintech), mouse anti-lamin B1 (1:500, Santa Cruz), rabbit anti-cyclin B1 (1:500, Santa Cruz), rabbit anti-HMGB1 (1:500, Abcam), mouse anti-PARP1 (1:500, BD Biosciences), mouse anti-p53 (1:250, Santa Cruz) and mouse anti-GAPDH (1:50000, Sigma) as a loading control. Proteins were detected after 1h of incubation at RT with appropriate horseradish peroxidase-conjugated secondary antibody (1:2000, Dako) using an ECL system (GE Health Care) according to the manufacturer’s instructions.

### Analysis of senescence-associated secretory phenotype (ELISA assay)

After 24-hours of culture medium from cells were collected and analyzed for the presence of secreted proinflammatory factors, namely interleukin 6 (IL-6), interleukin 8 (IL-8) according to a manufacturer’s protocol (R&D Systems). The level of secreted factors was determined with the use of standard curve and normalized to the number of the cells.

### Statistical analysis

Statistical analysis was performed using GraphPad Prism (San Diego, California, USA). Analysis of sample distribution was performed using the D'Agostino & Pearson normality test. The One-way ordinary ANOVA or Kruskal-Wallis test with FDR correction (two-stage step-up method of Benjamini, Krieger and Yekutieli) for multiple comparison was applied. Graphs present mean ± SEM, the number of independent biological replicates (N) is given in the legend of each figure for the specific analysis presented. A value of p < 0.05 was considered significant (*p < 0.05, ** p < 0.01, *** p < 0.001, **** p < 0.0001).

## RESULTS

### Discrimination of proliferating, quiescent and senescent cells on the basis of selected parameters

We analysed selected and predefined markers to differentiate proliferating cells, temporally growth-arrested cells and senescent cells. The analysis was performed on VSMCs, and the results were verified in preadipocytes and fibroblasts.

To induce senescence, proliferating VSMCs were treated with low, nontoxic concentrations of doxorubicin (DOX) or hydrogen peroxide (H_2_O_2_) and analysed 3 and 7 days after induction. The doses of doxorubicin and H_2_O_2_ have been selected to achieve effective growth inhibition without toxic effect. We have successfully used this experimental model in our previous studies [21, 23, 24]. Cells undergoing temporary growth arrest (quiescence) were obtained by culturing the cells in medium without FBS for 7 days (serum-starved cells, Q(s)); alternatively, the cells were cultured for 8 days without passaging until they reached full confluence (contact-inhibited cells, Q(i)). Control, proliferating cells were collected 3 days after plating (24, 48 and 72 hours) to assess the potential changes that are correlated with the growth dynamics of cultured cells.

To compare the proliferation potential of cells that were induced to senescence, quiescent cells and cells that were cultured under standard conditions, we analysed cell numbers, BrdU incorporation, Ki67 expression and cell cycle progression. The number of cells on days 3 (median=0.3 million cells, mean=0.313, SD±0.064, p> 0.958) and 7 (median=0.3 million cells, mean=0.296, SD±0.025, p>0.959) after DOX or H_2_O_2_ treatment (day 3, median=0.312 million cells, mean=0.310, SD±0.072, p>0.958; day 7, median=0.288 million cells, mean=0.310, SD±0.061, p>0.959) did not change and remained similar to the number of cells counted at the day of the treatment (24 h, median=0.3 million cells, mean=0.285, SD±0.041), indicating that the cells stopped proliferating immediately and no toxic effect of compounds was observed since no decrease in the number of cells that were induced to senescence was observed. The number of cells that were cultured for 7 days in serum-free medium (Qs) slightly, but not significant increased (median=0.71 million cells, mean=0.88, SD±0.286, p=0.958) compared to the number of proliferating cells that were collected after 48 hours (median=0.65 million cells, mean=0.665, SD±0.15), indicating that the proliferation of these cells was effectively limited, whereas the number of cells that were cultured under standard conditions doubled daily (Fig. 1A). The number of contact-inhibited confluent cells (Qi) reached its highest value after seven days of culture (median=2.125 million cells, mean=2.4, SD±0.897), which is twice the number of cells collected after 72 hours of culture (median=1.225 million cells, mean=1.22, SD±0.375). Statistical analysis showed significant differences in number of proliferating (24h) and quiescent cells (Qs, p=0.036; Qi, p=0.0037). BrdU incorporation was analysed to estimate the number of cells that underwent DNA replication. Within the first 24 hours of culture, 63.8 % of proliferating cells underwent DNA replication (median=63.7%, mean=63.8%, SD±3.44), and in this number increased in the following 48 (median=82.85%, mean=82.85%, SD±2.25) and 72 hours (median=75.15%, mean=75.65%, SD±3.8). Few of the cells that were treated with doxorubicin underwent DNA replication after 7 days (median=0%, mean=0.025%, SD±0.05, p=0.0002), whereas only 5.95% (median, mean=8.613%, SD±7.98, p=0.0296) of the cells treated with H_2_O_2_ for 7 days were able to incorporate BrdU. A decrease in the ability to synthesize DNA was also observed in quiescent cells (Qs, median=10.28%, mean=10.85%, SD±4.08, p=0.052; Qi, median=30.6%, mean=31.06%, SD±4.09, p=0.256) compared with proliferating cells (24h) (Fig. 1B, D.).

**Figure 1.**
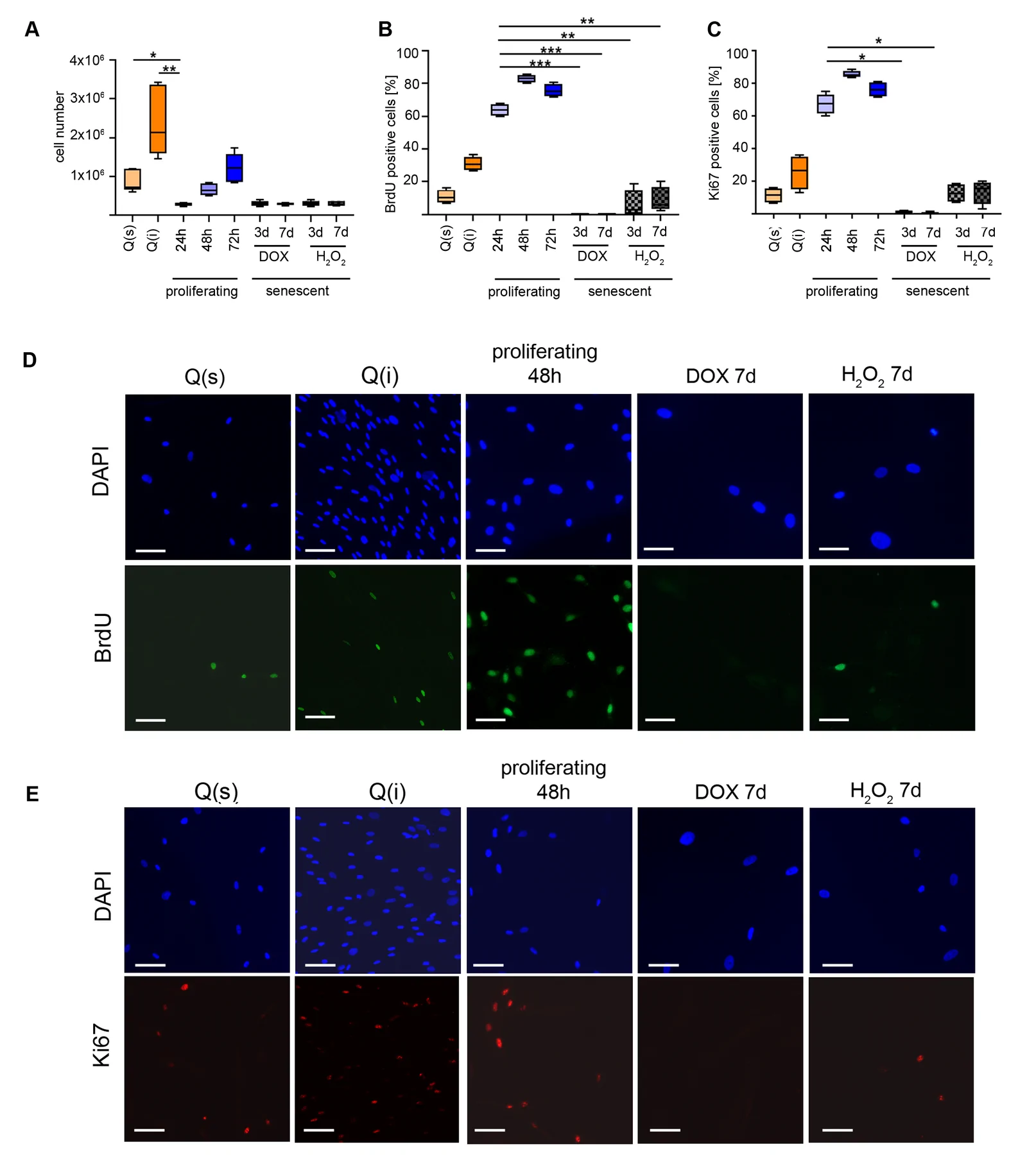
**Analysis of proliferation markers in VSMCs**. Comparison of cell number (A), BrdU incorporation (B) and Ki67 expression (C) in quiescent, proliferating and DOX- or H_2_O_2_-treated VSMCs. To obtain quiescent cells, cells were cultured for 7 days without replating until they stopped proliferating due to contact inhibition (Qi) or under starvation conditions (cultured in serum-free medium, Qs). Proliferating cells were analysed 24, 48 or 72 hours after plating. To induce senescence, cells were treated with low, nontoxic doses of doxorubicin (DOX, 150 nM) or H_2_O_2_ (150 µM) and analysed at the indicated times. The statistical significance of differences between proliferating (24h), quiescent and senescent cells for all the measurements presented in (A, B, C) were analysed using Kruskal-Wallis test with FDR correction (two-stage step-up method of Benjamini, Krieger and Yekutieli) for multiple comparison, * p<0.05, ** p< 0.01, ***p<0.001, ****p<0.0001; data are presents as median, min to max, N=5. Representative images of immunocytochemical detection of BrdU-incorporating cells (D) and Ki67-positive cells (E) are shown.

Thereafter, we analysed the protein level of Ki67, which is a marker of cells progressing through the cell cycle, and its absence indicates cell cycle arrest. The majority of cells that were cultured under standard conditions (proliferating cells) expressed Ki67. The number of Ki67-positive cells increased to 85.45% (median, mean=85.78, SD±4.48) in the first 2 days of culture (48h). In contrast, cells that were treated with doxorubicin (day 7, median=0, mean=0.4, SD±0.8, p=0.0124) or hydrogen peroxide (day 7, median=15.8%, mean=13.78%, SD±4.42, p=0.238) and quiescent cells (Qs, median=11.85%, mean=11.64%, SD±4.42, p=0.2; Qi, median=26.8%, mean=25.79%, SD±10.36, p=0.458) exhibited a decrease in Ki67 expression (Fig. 1C, E.).

**Figure 2.**
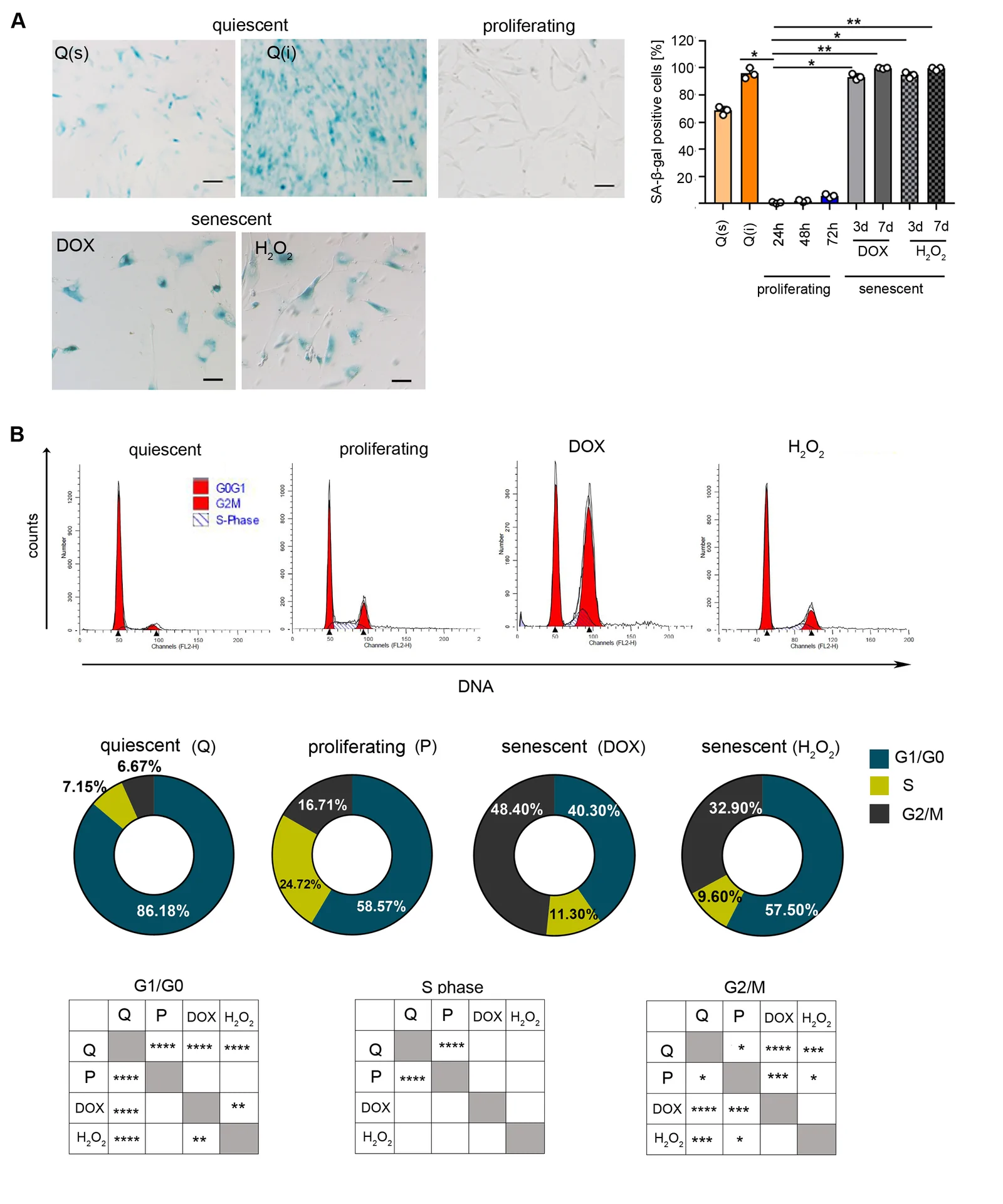
**SA-β-gal activity and cell cycle analysis in proliferating, quiescent and senescent VSMCs**. (**A**) Analysis of SA-β-gal activity with a cytochemical test was performed and representative images of proliferating, quiescent and DOX- or H_2_O_2_-treated cells are shown on the left. Graph presents the mean ± SEM percentage of positive (blue) cells, N=3. (**B**) Representative histogram of DNA content measurement by flow cytometry (upper panel). Mean percentages of cells in the G1/G0, S or G2/M phases of the cell cycle in the quiescent (Qi and Qs), proliferating (24 and 48 and 72 hours of culture) and senescent (DOX- and H_2_O_2_-induced) groups are presented as donut graphs (middle panel). The statistical significance of differences of cells in G1/G0, S and G2/M phases between quiescent, proliferating, DOX- or H_2_O_2_-treated VSMCs was analysed using Kruskal-Wallis test with FDR correction (two-stage step-up method of Benjamini, Krieger and Yekutieli) for multiple comparison, *p<0.05, **p< 0.01, ***p<0.001, ****p<0.0001; data are presents as mean, N=5. Statistically significant differences in each phase of the cell cycle between the groups are presented in the tables (lower panel).

We estimated the level of SA-β-galactosidase activity, which is a basic, although not specific, marker of cellular senescence. Accordingly, almost all of the DOX- (median=93.2%, mean=93.33%, SD±2.2, p=0.5) and H_2_O_2_-treated cells (median=94.8%, mean=94.6%, SD±2.2, p=0.0038) exhibited increased SA-β-galactosidase activity after only 3 days of treatment. Interestingly, increased activity of β-gal, which is usually correlated with senescence, was also observed in quiescent cells in both the confluent (median=95.0%, mean=95.77%, SD±3.9, p=0.024) and serum-starved (median=68.9%, mean=68.47%, SD±2.97, p=0.240) groups (Fig. 2A).

Finally, we compared the cell cycle distributions of the proliferating, quiescent and senescent cell populations. Quiescent cells accumulated mainly in the G0/G1 phase (86.18%), whereas cells that were induced to undergo senescence accumulated in the G0/G1 phases (DOX - 40.3%, H_2_O_2_ - 57.5%) and G2/M phase (DOX - 48.4%, H_2_O_2_ - 32.9%). Statistical analysis showed that the number of cells in the G2/M phase and G0/G1 phase can distinguish senescent and proliferating cells from quiescent cells, whereas proliferating cells can be distinguished from senescent cells solely by the difference in the number of G2/M phase cells (Fig. 2B). Importantly, cell type and senescence trigger-specific variability was observed with respect to cell cycle distribution (Supp. Fig.2) Moreover, based on cell cycle analysis, we also noted that the percentage of subG1 fraction in cells treated with DOX or H_2_O_2_ did not exceed 5% in all experiments, which further confirms the lack of toxic effects of this treatment (not shown).

To compare proliferating and nonproliferating cells (quiescent and senescent cells), the levels of selected proteins were analysed. To this end, we choose proteins that are often used to identify senescent cells, namely, Lamin B1 (nuclear envelope protein), HMGB1 (High mobility group box 1, a chromatin protein), p53 (DNA damage response protein), p21 and p16 (cell cycle inhibitors). We also included two other proteins in the analysis: Cyclin B1 and poly (ADP-ribose) polymerase 1 (PARP1). Cyclin B1 is involved in regulating mitosis and can serve as a marker of the mitotic potential of a cell population. PARP1 is involved in DNA repair and has recently been shown to be downregulated in senescent fibroblasts [25]. Moreover, our studies revealed a significant decrease in PARP1 expression in senescent VSMCs (unpublished data).

By the third day after the induction of senescence, we observed a decrease in Lamin B1, HMGB1 and PARP1 protein levels compared with those in proliferating cells. Moreover, the levels of these proteins decreased significantly even further by day 7. Interestingly, we observed an increase in the Lamin B1 and PARP1 levels in proliferating cells over subsequent days of culture. The protein levels of HMGB1, Lamin B1 and PARP1 did not change in quiescent cells and remained the same as those in proliferating cells. Importantly, we did not observe PARP1 cleavage in cells treated with DOX and H_2_O_2_, indicating a lack of caspase activation and apoptosis induction in senescent cells (Fig. 3A).

The level of Cyclin B1 was the highest in proliferating cells at the beginning of culture (24 hours after plating), but then it gradually decreased after 48 and 72 hours. Quiescent cells showed significantly reduced levels of Cyclin B1, reflecting the lack of mitotic activity in this population. Similarly, cells that were induced to undergo senescence lost Cyclin B1 expression after 3 days of treatment, and the Cyclin B1 protein remained almost undetectable a few days later (Fig. 3A).

The long-lasting upregulation of the p21 and/or p16 proteins is considered the fundamental marker of cellular senescence. p21 expression is induced by the p53 protein in response to DNA damage, and p16 leads to the hypophosphorylation of the Rb protein. Both p21 and p16 cause cell cycle arrest, which is the main feature of senescent cells. p53 protein expression reached a high level in DOX-treated cells, whereas H_2_O_2_ caused moderate induction of p53 protein expression only 7 days after treatment. Notably, the level of p53 was also increased in proliferating cells (48 hours) and reached the level observed in senescent cells (Fig. 3A). Moreover, the level of p53 in quiescent cells was comparable to that observed in proliferating cells (24 and 72h). Similar to p53, p21 protein expression was induced in senescing cells, and its level was greater on day 3 than on day 7. Interestingly, p21 was also upregulated in proliferating cells on subsequent days of culture, reaching a level that was comparable to that observed in senescent cells. Quiescent cells did not differ significantly from proliferating and senescent cells in terms of p21 expression. In contrast to proteins involved in the DNA damage response pathway (p53 and p21), p16 was significantly increased only in quiescent VSMCs (Qs), whereas the p16 levels remained unchanged in proliferating and senescent cells (Fig. 3A).

**Figure 3.**
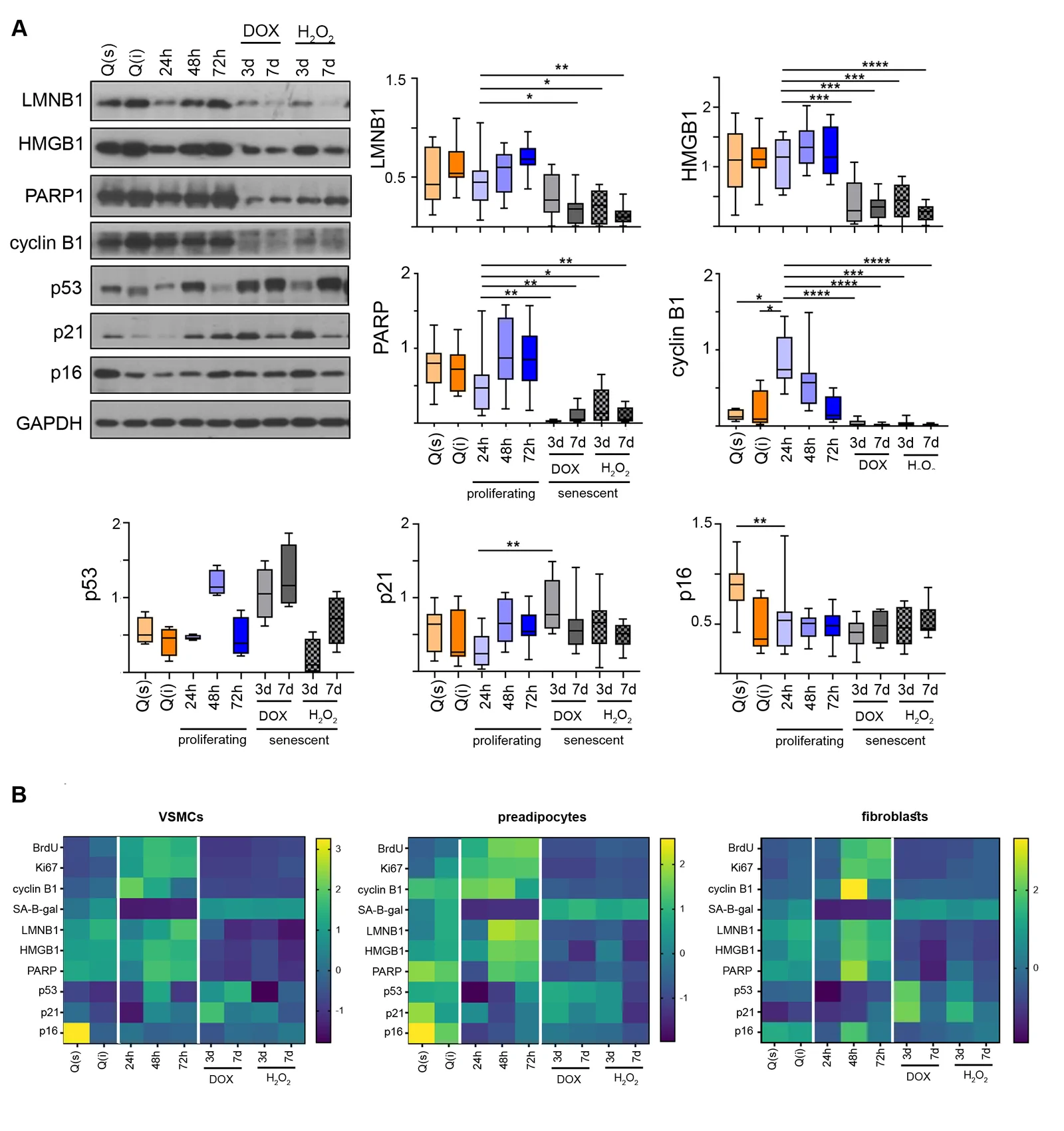
**Expression of selected proteins in quiescent, proliferating and senescent VSMCs**. (**A**) Western blotting analysis of Lamin B1 (LMNB1), HMGB1, PARP1, Cyclin B1, p53, p21 and p16 expression. The level of GAPDH served as a loading control. Representative blots are shown (left). Densitometric analysis was performed, the data for each protein were normalized to the GAPDH level, and the results are presented in the graphs (median, min to max), N=8. Statistical analysis was performed using one-way ANOVA corrected for multiple comparison. The statistical significance of differences between proliferating (24h), quiescent and senescent cells for each protein was analysed using one-way ANOVA with FDR correction (two-stage step-up method of Benjamini, Krieger and Yekutieli) for multiple comparison, *p<0.05, ** p< 0.01, ***p<0.001, ****p<0.0001. (**B**) Comparative analyses of the expression levels of proliferation and senescence markers in VSMCs, preadipocytes and fibroblasts are presented as heatmaps. The analysis was carried out via Z standardization. Graphs presenting data for fibroblasts and preadipocytes, which are included in heatmaps, are shown in Supplementary Figs. 1, 2, 3 and 4.

To define a set of markers that best distinguish among proliferating, quiescent and senescent cells, we analysed all the aforementioned parameters in two additional cell types, namely, preadipocytes and fibroblasts. The pattern of changes in protein levels was comparable to that observed in VSMCs. Few differences were observed, namely, p53 and p21 levels were clearly higher 3 days after DOX or H_2_O_2_ treatment of fibroblasts than in proliferating or quiescent fibroblasts. Moreover, the level of Cyclin B1 in fibroblasts remain at comparable, low level in quiescent and senescent cells whereas the increase of the expression of this protein was observed in proliferating cells only after two days of culture followed by a decrease (Supplementary Figs. 3 and 4).

Heatmaps (Fig. 3B). visualize the comparison of levels of all analysed biomarkers in VSMCs, preadipocytes and fibroblasts. Detailed data for all the measurements are presented in Supplementary Figs. 1, 2, 3 and 4

On the basis of this analysis, we identified two groups of markers. One group consists of BrdU, Ki67 and SA-β-gal, whose levels are similar in growth-arrested (senescent and quiescent) cells but significantly different in proliferating cells. The relative level of Cyclin B1 analysed in proliferating, quiescent and senescent cells differed slightly between these groups depending on the cell type and remained at a similar level in quiescent and senescent cells (VSMCs, fibroblasts). The other group includes the Lamin B1, HMGB1 and PARP1 proteins, the levels of which are similar in proliferating and quiescent cells but significantly different in senescent cells. Interestingly, the protein expression levels of p53, p21 and p16 are variable and unstable depending on the condition and cell type. For this reason, the p53, p21 and p16 proteins appear to be less reliable markers of senescence.

**Figure 4.**
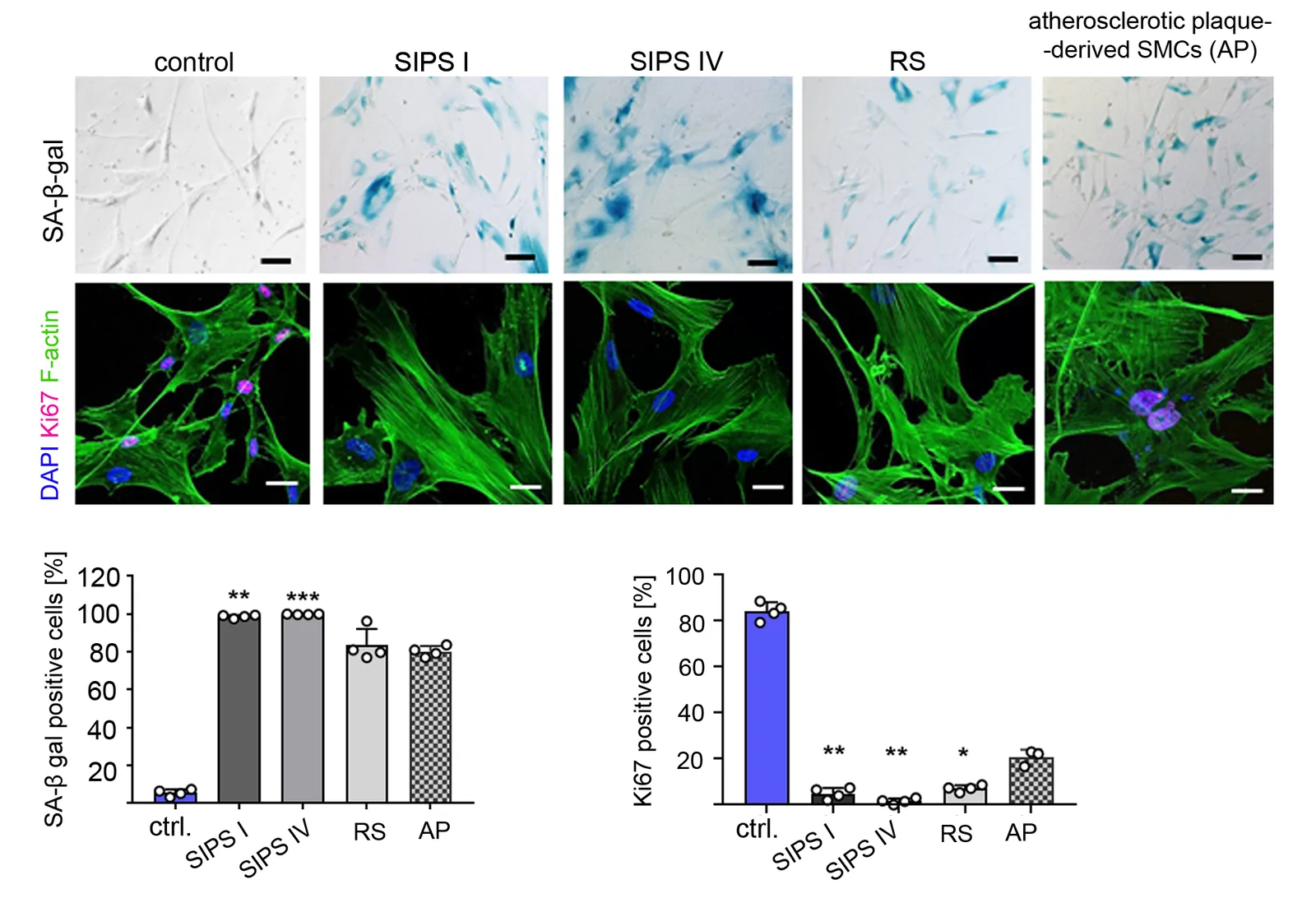
**Analysis of SA-β-gal activity and Ki67 expression in VSMCs that underwent senescence *in vitro* (SIPS and RS) or *in vivo* (atherosclerotic plaque-derived cells, AP)**. Representative images showing SA-β-gal activity and Ki67 expression in control and senescent VSMCs; scale bar=20 µm (upper panel). To induce senescence, proliferating VSMCs (ctrl.) were treated with H_2_O_2_ and cultured for 1 week (SIPS I) or 4 weeks (SIPS IV). Additionally, cells were cultured for a few months until they reached a state of replicative senescence (RS). Plaque-derived cells (AP) were obtained as described in the Materials and Methods section and analysed thereafter. Graph presents mean percentage ±SEM (N=4) of SA-β-gal-positive and Ki67-positive cells in each experimental group. The statistical significance of differences between control and SIPS I, SIPS IV, RS or AP cells were analysed using Kruskal-Wallis test with FDR correction (two-stage step-up method of Benjamini, Krieger and Yekutieli) for multiple comparison, * p<0.05, ** p< 0.01, ***p<0.001 (lower panel)

### Comparison of expression of selected markers in VSMCs undergoing senescence *in vitro* and *in vivo*

Based on the results of previously conducted analyses, the expression of selected markers in VSMCs that underwent senescence in vitro and in vivo was compared. In vitro senescence was studied using a model of H_2_O_2_-induced senescence (stress-induced premature senescence, SIPS) and a model of replicative senescence (RS), i.e., cells that gradually lost proliferative potential over 3 months of culture. Furthermore, two time points were distinguished for SIPS: 7 days post-treatment (SIPS I, early senescence) and 4 weeks post-treatment (SIPS IV), reflecting the late phase of senescence. To analyse cells that underwent senescence in vivo we isolated VSMCs from atherosclerotic plaques collected from patients during carotid endarterectomy.

The level of senescence markers in SIPS I, SIPS IV, RS and atherosclerotic plaques-derived VSMCs were compared with low-passage VSMCs obtained from healthy aortas. Analysis of the number of SA-β-gal-positive and Ki67-negative cells revealed that more than 90% of the SIPS and RS cells were growth-arrested. The percentages of SA-β-gal (+) and Ki67(-) cells obtained from the plaques were lower, but they still constituted the majority of the total population (Fig. 4).

**Figure 5.**
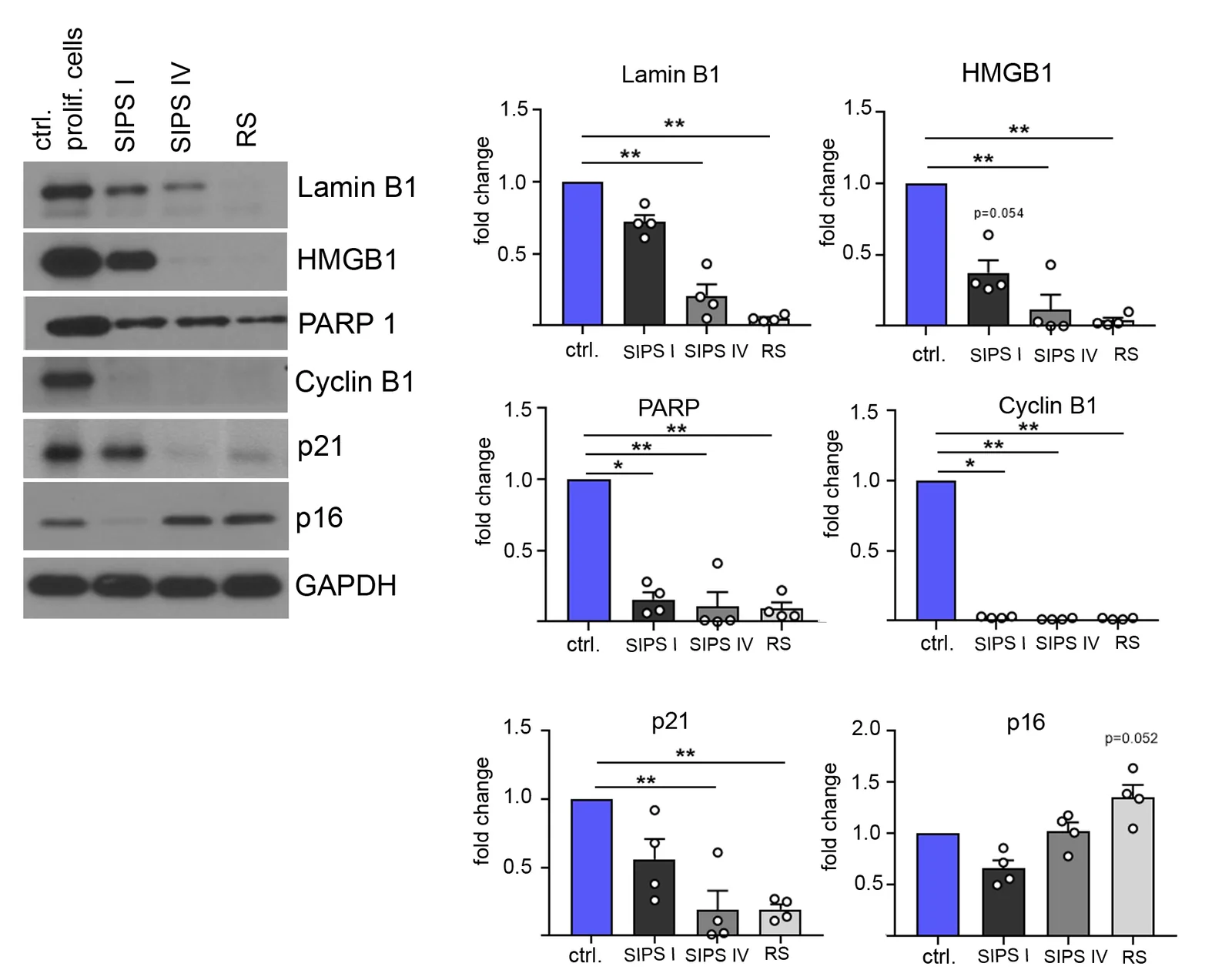
**Comparison of the levels of selected protein markers in VSMCs that underwent senescence *in vitro* (SIPS and RS)**. Proliferating VSMCs (control cells) were induce to senescence by H_2_O_2_-treatment and cultured for 1 week (SIPS I) or 4 weeks (SIPS IV). Additionally, cells were cultured for a few months until they reached a state of replicative senescence (RS). The levels of selected proteins in VSMCs that were induced to undergo senescence *in vitro* were analysed by Western blotting. Representative blots are presented (left panel). Densitometric measurements from 4 independent experiments were performed. Data for each protein were normalized to the level of GAPDH and presented as a relative value to the level of given protein in control cells, means ±SEMs, N=4. The statistical significance of differences between control and SIPS I, SIPS IV or RS cells were analysed using Kruskal-Wallis test with FDR correction (two-stage step-up method of Benjamini, Krieger and Yekutieli) for multiple comparison, * p<0.05, ** p< 0.01.

Western blotting analysis revealed a significant reduction in the level of Cyclin B1 in all three groups of cells that were induced to undergo senescence: premature senescence at the early (SIPS I) and late (SIPS IV) stages, as well as replicative senescence. A significant decrease in the protein levels of Lamin B1, HMGB1 and PARP1 was also observed in these cells (Fig. 5). Previously obtained results revealed a gradual decrease in HMGB1 and Lamin B1 expression after 3 and 7 days of doxorubicin or H_2_O_2_ treatment. A progressive decrease in the levels of these proteins was also observed in cells in the late stage of senescence (SIPS IV) compared with cells in SIPS I and control cells. The lowest levels of Lamin B1 and HMGB1 were detected in cells undergoing replicative senescence (RS). Interestingly, a significant decrease in p21 protein expression and increase in p16 expression were also observed as the cells progressed to the late stage of senescence (SIPS IV), with the lowest p21 expression and highest p16 expression observed in RS cells (Fig. 5).

A similar analysis of selected proteins was performed in smooth muscle cells that were isolated from atherosclerotic plaques from patients. The protein levels in these cells were compared with those in smooth muscle cells derived from normal, healthy aortas (control). A significant decrease in Lamin B1, HMGB1, PARP1 and Cyclin B1 levels was observed in cells that were isolated from atherosclerotic plaques. Interestingly, an increase in the p21 levels was observed in cells that were isolated from atherosclerotic plaques, which is opposite of the trend that was observed in cells that underwent senescence *in vitro*. In contrast, the increase in the p16 level in plaque-derived cells was similar to that in cells in late-stage senescence (Fig. 6A). Analyses of protein marker levels revealed heterogeneity in VSMCs obtained from atherosclerotic plaques. Interestingly, the results obtained for individual atherosclerotic plaques indicated a high (0.7≤ r_xy_ <0.9) or very high (0.9≤ r_xy_ <1) degree of correlation between the levels of Lamin B1, HMGB1, and Cyclin B1, whereas the correlation coefficients between p21, p16 and HMGB1, Lamin B1, and Cyclin B1 were lower and ranged (0.38≤ r_xy_ <0.58). On the other hand, PARP1 level, although significantly decreased in VSMCs derived from atherosclerotic plaques, was found to be relatively weakly correlated with cyclin B1 level (r_xy_=0.31) and moderately correlated with other senescence markers - Lamin B1, HMGB1, p21, p16 (0.40 ≤ r_xy_ <0.59) (Fig.6B).

**Figure 6.**
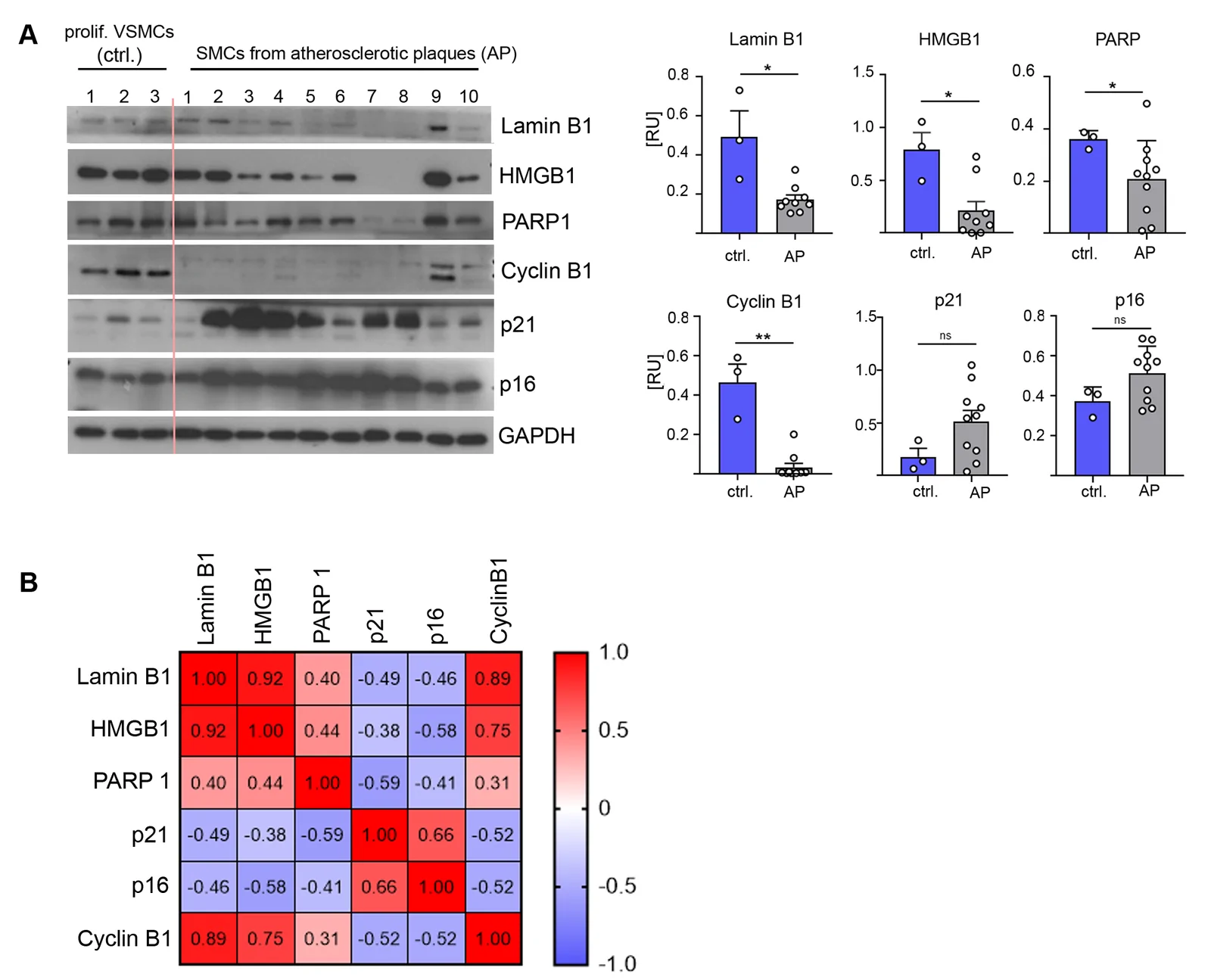
**Analysis of selected protein markers of senescence in atherosclerotic plaque-derived cells (AP)**. (**A**) The levels of selected proteins in cells derived from atherosclerotic plaques (AP) were analysed using Western blotting. VSMCs derived from aortas from 3 different donors (N=3), at early passage (p≤6), constitute the control (ctrl.) and were compared with AP-derived VSMCs obtained form 9 patients (N=9). Blots presenting the level of each protein is shown (left panel). The graph presents the results of densitometric measurement following Western blotting analysis, mean ±SEM. Statistical analysis was performed with Mann Whitney test, *p<0.05, ** p< 0.01. (**B**) Heat map showing Pearson correlation coefficients between analyzed proteins.

We then analysed the levels of representative SASP factors, IL-6 and IL-8, secreted by vascular smooth muscle cells (VSMCs). As expected, senescent cells (SIPS I and SIPS IV) secreted increased amounts of interleukins compared with proliferating, control, and quiescent cells. Interleukin levels tended to increase with the development of the senescent phenotype over time (SIPS IV vs. SIPS I). Importantly, increased IL-6 and IL-8 secretion was found in vascular smooth muscle cells (VSMCs) derived from atherosclerotic plaques (APs). It is worth noting that AP-derived cells secrete more IL-8 than IL-6 and more than cells undergoing senescence in vitro (SIPS I, SIPS IV) (Fig. 7).

**Figure 7.**
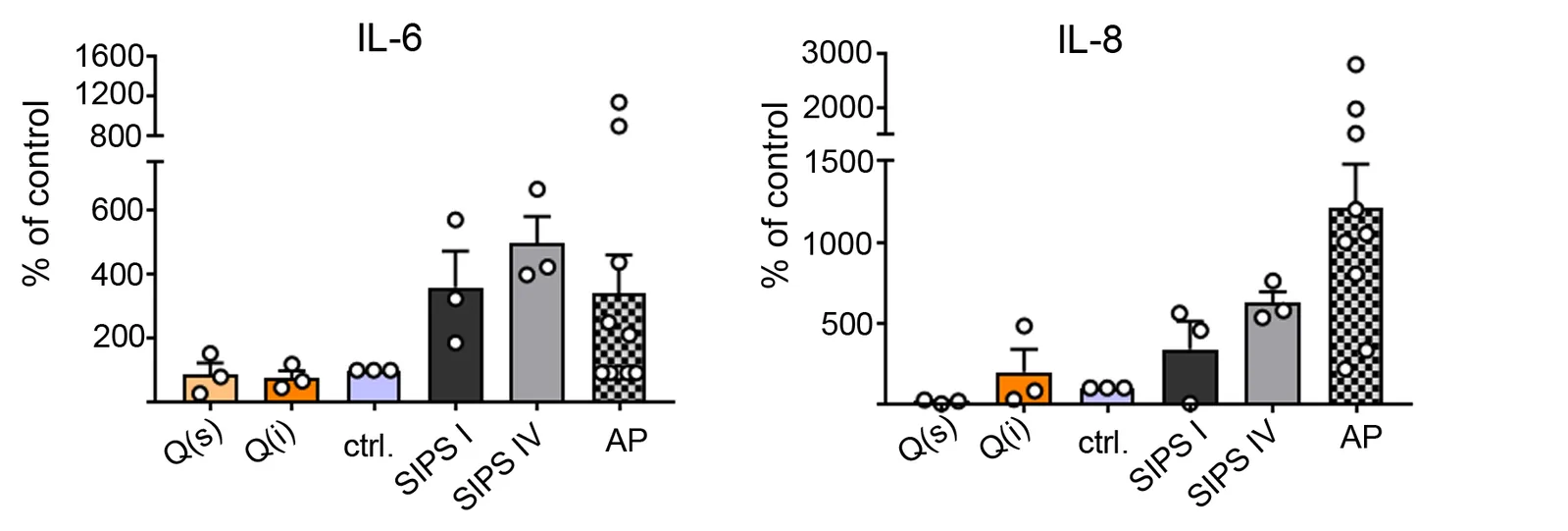
**Estimation of the levels of representative SASP factors (IL-6 and IL-8) secreted by quiescent, senescent and atherosclerotic plaque-derived VSMCs**. The level of IL-6 and IL-8 were measured in condition medium collected after 24h of culture with quiescent, proliferating (control), senescent (SIPS I and IV) and AP-derived VSMCs using Elisa assay. The amount of cytokines secreted was normalized to cell number and presented on the graph as relative change from control, mean ±SEM, N=3 (for Q, ctrl, SIPS cells) or N=9 (for AP cells).

## DISCUSSION

To date, no single universal marker can be used to identify senescent cells, and a combination of different biomarkers has been widely used in attempts to distinguish senescent cells from other primary nonsenescent cells. Although a number of biomarkers related to the molecular, functional and morphological features of senescent cells have already been described [10], the suitability of individual markers for identifying senescent cells varies depending on the particular stress to which a cell is exposed, the type of cell, the time that has passed since senescence was induced, the persistence of marker expression, and the sensitivity of marker detection methods. Using genetic analysis, several studies have shown variability in the senescent phenotype at the single-cell [26, 27] or whole-transcriptome level [28]. An important limitation of transcription-based studies is that mRNA transcript levels may not reflect the steady-state protein levels. Moreover, many studies that have characterized senescent cells used proliferating cells as controls, which might sometimes lead to the misinterpretation of results. The aim of our study was to define a set of markers that would enable the distinction between proliferating, senescent, and temporarily growth-arrested (quiescent) vascular smooth muscle cells (VSMCs), i.e., three different cell populations occurring in different proportions in the tissue. We then verified the usefulness of defined *in vitro* induced senescence markers for assessing *in vivo* senescence of VSMCs derived from atherosclerotic plaque. Although senescence of VSMCs in atherosclerotic plaques has been reported previously, to our knowledge this is the first time that this cell type has been studied in detail, allowing for the estimation of a set of senescence markers in individual plaques. We analyzed commonly used, but also less recognized, easy-to-analyze markers of senescence. Those included growth arrest markers like BrdU incorporation and Ki67 expression but also Cyclin B1 level and cell cycle arrest. Cyclin B1 is a key cell cycle protein that regulates transition from G2 to M phases [29], which decreased level has also been correlated with the senescence [30, 31]. In addition, we analyzed proteins involved or correlated with the induction and establishment of senescence, such as p53, p21 - proteins belonging to the DNA damage response pathway, p16 - an inhibitor of CDK4/6 commonly considered a marker of senescent cells, especially in *in vivo* studies [10], and nuclear proteins such as Lamin B1, HMGB1 and PARP1.We defined signatures of quiescent, proliferating and senescent cells using VSMCs that were induced to undergo senescence by treatment with doxorubicin or H_2_O_2._ To confirm the obtained results, we also analysed other primary cell types - fibroblasts and preadipocytes, which undergo senescence or quiescent after the same treatment. Our study revealed that the most consistent markers of senescence both *in vitro* and *in vivo* of VSMCs, are nuclear proteins, namely, Lamin B1 (nuclear envelope protein), HMGB1 (High mobility group box 1, a chromatin protein), and PARP1 (poly (ADP-ribose) polymerase 1, DNA repair protein). Downregulation of Lamin B1 in senescent human and mouse fibroblasts was described for the first time by Freud et al. (2012) [32], who showed that lamin B1 mRNA level declined at senescence due to decrease in lamin B1 mRNA stability but also due to autophagic degradation [33]. Shah et al. (2013)[34] showed that Lamin B1 reduction in proliferating cells triggers senescence and postulated that lamin B1 down-regulation in senescence is a key trigger of global and local chromatin changes that impact gene expression. It was also demonstrated that loss of lamin B1 facilitates the detection and quantification of senescent cells upon chronic UV-exposure and skin regeneration *in vivo* [35]. HMGB1 operates as a dual-function protein with distinct nuclear and extracellular activities. It binds DNA in a sequence-independent manner and alters chromatin architecture, allowing transcription factor binding. Upon stress HMGB1 translocates to the cytoplasm and is secreted into the extracellular space, where it interacts with receptors to induce inflammatory responses [36]. HMGB1 acts as RNA-binding protein (RBP) thus regulating availability of SASP relevant transcripts [37]. Unlike Lamin B1 and HMGB1, the role of PARP1 in senescence is less recognized. Recently, Neheme at al. (2024) [25] have shown, using publicly available datasets, that PARP1 is a consistently downregulated gene during senescence and validated this finding experimentally in senescent fibroblasts [25]. Moreover, they demonstrated that blocking PARP1 activation in cells exposed to high oxidative stress promotes conversion of cell death into senescence. Unlike aforementioned nuclear proteins, proteins involved in signalling pathways commonly associated with the induction and establishment of senescence, such as p53, p21 and p16, exhibited ambiguous expression pattern *in vitro* model of VSMCs senescence. These findings suggests that changes in the levels of these proteins are much more subtle and/or dependent on time and cell type, and these proteins cannot always be used to confirm the senescent phenotype. Unexpectedly, we observed statistically significant increase of p16 only in quiescent VSMCs (serum-starved cells) and preadipocytes (serum-starved and contact-inhibited cells). Although p16 is widely considered to be related to senescence, increased level of this protein was also noticed in fibroblasts and mammary cancer cells induced to quiescence by serum starvation [38]. We also observed transient upregulation of p53 in proliferating VSMCs and preadipocytes (48h or 72 after plating respectively) up to level observed in DOX-treated cells. Although the accumulation of p53 in proliferating cells was surprising, Loewer et al. (2010) [39] had already described this phenomenon. Namely, he demonstrated the presence of p53 pulses in non-stressed cells, that were as strong as the pulses in irradiated cells. However, in the absence of sustained damage, post-translational modifications keep p53 inactive. In other words, transient DNA damage triggers p53 accumulation, while sustained damage signalling is necessary to change the p53 modification state from inhibitory to active [39]. It should also be noted that even within the proliferating primary cells population, there is still a fraction of growth-arrested cells, as evidenced by the lack of BrdU incorporation and lack of Ki67 expression. These cells may have acquired spontaneous DNA damage, leading to the observed upregulation of p21 in proliferating VSMCs (48 hours *versus* 24 hours after plating).

The cell proliferation markers such as, BrdU incorporation, Ki67 and Cyclin B1 expression, though they unambiguously confirmed the growth-arrested state of senescent cells, did not unequivocally discriminate between quiescent and senescent cells. While the level of Cyclin B1 remained low in quiescent and senescent cells, which corresponded to low levels of BrdU incorporation and low Ki67 expression, significant changes in the level of this protein were also observed in the subsequent days of culture of the proliferating cell population. Although we did not investigate this phenomenon in detail, we speculate that the observed changes in Cyclin B1 levels may reflect changes in the mitotic activity of cells after plating. Cyclin B1 is involved in the regulation of mitosis, and its levels fluctuate throughout the cell cycle, peaking in late G2 and mitosis and then decreasing in G1 [40, 41]. Therefore, elevated Cyclin B1 levels may result from an increased number of mitotic and/or late S and G2 cells that peaks 24-48h after plating depending on cell type. It should be noted that the temporal increase in Cyclin B1 expression in proliferating cells did not correlate with mitotic arrest, as we did not observe accumulation of mitotic cells under the microscope, but also in the cell cycle analysis (not shown).

The accumulation of cells in the G2/M phase (DNA 4N cells) is the most reliable marker of proliferation, which allows distinguishing temporarily growth-arrested or proliferating cells from senescent cells. However, this may apply only to cells undergoing stress-induced premature senescence, not replicative senescence, during which cells arrest predominantly in the G1/G0 phase [24]. On the other hand, SA-β-gal activity is the gold standard senescence marker; however, although an increased in this activity is necessary to confirm senescence, this marker is unspecific, and as such, it could lead to false-positive results, especially in cells that are subjected to improper cell culture conditions [42, 43].

Cellular senescence is a process during which the phenotype of a cell evolves and changes with time [28]. Accordingly, we analysed senescent VSMCs after prolong culture (up to 4 weeks after induction of senescence) and observed that the levels of some senescence marker proteins, such as Lamin B1 and HMGB1, decrease as cells progress to the later stage of senescence (SIPS I *versus* SIPS IV). We also included VSMCs that underwent replicative senescence (RS), which reflects a more physiological process of cellular ageing that progress gradually over months, in the analysis. Interestingly, VSMCs that underwent RS presented the lowest levels of Lamin B1 and HMGB1, suggesting that both the SIPS IV population and the RS population consisted of cells that presented the most ‘mature’ senescence phenotype; these results indicated that the time spent in a state of growth arrest influences the expression of at least some markers. A similar conclusion was drawn from studies performed by Ashraf et al. [44], who reported that the intensity of multiple senescence biomarkers is graded rather than binary and reflects the duration of cell cycle arrest. He analysed the correlation between the expression of selected senescence markers and the time spent in growth arrest at the single-cell level. Among the eight parameters, including SA-β-gal, 53BP1, p21, IL8, Lamin B1, LAMP1, and the cytoplasmic and nuclear areas, p21 and Lamin B1 exhibited the strongest ability to discriminate between senescent and presenescent (slow-cycling) cells. Notably, in our studies, the level of p21 decreased in late senescent cells, suggesting that p21 is an early marker related to the time-limited phase of senescence induction, which is consistent with studies by Ashraf, which were performed within a time scale of up to 4 days. Consistent with our observations, Garrido et al. (2022) [45] revealed that p21 expression in VSMCs induced to senescence by doxorubicin increase shortly after treatment and then decrease. Therefore, they postulated that upregulation of p21 during senescence induction should be considered rather as a marker of DNA damage. In contrast to p21, p16 was upregulated only in late senescent VSMCs (SIPS IV) and in VSMCs that underwent replicative senescence (RS). Although the p16 protein is most commonly considered a senescence marker, especially in *in vivo* studies, it is not expressed in all senescent cells, and the use of this marker may lead to an underestimation of the number of senescent cells. Notably, p21 and p16 are both cell cycle inhibitors. However, on the basis of our observations, neither is correlated with cell cycle arrest markers, such as BrdU incorporation and Ki67 expression, at least over a longer time period. Our studies are consistent with the view that the p16 protein is a marker of cells in later stages of senescence [45, 46]. Based on result of *in vitro* senescence studies, we explored the degree of senescence in VSMCs derived from atherosclerotic plaques. Matthews et al. [47] previously reported that, during atherosclerotic plaque development, aortic smooth muscle cells undergo both replicative and stress-induced senescence, mostly due to increased ROS levels in plaques. Therefore, we isolated VSMCs from atherosclerotic plaques and assessed selected markers in cells that had undergone senescence *in vivo*. Since we did not passage VSMCs that were isolated from plaques but performed the analysis when only a sufficient number of cells had migrated from tissue explants, we assumed that this VSMC population reflects the characteristics of these cells under *in vivo* conditions. Indeed, the majority of plaque-derived VSMCs were positive for SA-β-gal and negative for Ki67. Furthermore, we observed significant changes in the levels of Lamin B1, HMGB1, and PARP1 comparable to those observed in cells undergoing senescence *in vitro*. Also, p16 expression was elevated, though not statistically significant, in plaque-derived VSMCs compering to proliferating cells. Interestingly, the protein expression of p21 showed an inverse trend when its levels in plaque-derived cells were compared with those in cells that were induced to undergo senescence *in vitro*. Since p21 is upregulated as a downstream target of the DNA damage response pathway, we hypothesize that increased levels of this protein in plaque-derived cells might reflect an increased degree of DNA damage that the cells acquire in the plaque microenvironment, mostly due to high levels of ROS. Oxidative stress is widely recognized as an important factor driving the development of atherosclerotic plaque [48]. Indeed, upregulation of ROS level as well as oxidative damage (8-oxo-G) was demonstrated in plaque-derived VSMCs. This changes were correlated with senescent phenotype of the cells [47]. This hypothesis is consistent with the concept of cellular ageing, that proceed cellular senescence. During the process of cellular ageing, the progression of internal changes, such as DNA damage, that occur in primary cells leads to the induction of cellular senescence [46]. Compared with cells undergoing *in vitro* senescence, tissue-derived cells are much more heterogeneous in terms of cellular age and represent a mixture of cells at different stages of senescence. Some of them are at early post-induction phase, correlated with an active DNA damage response and high levels of p21, whereas others have progressed to later stages of senescence characterized by lower levels of p21, analogous to that observed in VSMCs senescing *in vitro*. Therefore, cells in which the DNA damage response is activated and leads to p21 expression may be more numerous in plaque-derived VSMCs and coexist with cells that express senescence markers but low level of p21.

Collectively, our studies demonstrated heterogeneity in the expression of p21, p16, and PARP1 proteins in VSMCs derived from plaques from different donors, whereas Lamin B1 and HMGB1 levels remained relatively similar across tissue samples. Moreover, p21 and p16 showed only a moderate correlation with Cyclin B1 protein levels, a marker of mitotic activity in a given cell population. Furthermore, p21 and p16 levels do not correlate strongly with other markers of senescence, such as Lamin B1 and HMGB1. Since the increased expression of these two cell cycle inhibitors is restricted to different time windows in the development of the senescent phenotype, as discussed earlier [45, 46], the expression levels of Lamin B1 and HMGB1 have the highest predictive value for identifying senescent VSMCs in the atherosclerotic plaque.

In summary, we have defined a set of biomarkers that can confidently distinguish senescent cells from proliferating cells and cells undergoing temporal growth arrest (quiescence), regardless of the senescence trigger, cell type, or senescence phase (early or late), both *in vivo* and *in vitro*. We propose that combining analysis of the expression levels of these proteins with senescence markers specific to a given senescent cell type may increase the likelihood of accurately identifying senescent cells in a given tissue.

## Supplementary Materials

The Supplementary data can be found online at: www.aginganddisease.org/EN/10.14336/AD.2025.0687.

## Data Availability

The datasets generated during and/or analysed during the current study are available from the corresponding author upon reasonable request.
