# Supplementary material for: A Signature of Senescence Based on An Analysis of Selected Markers in Primary Vascular Smooth Muscle Cells Cultured In Vitro and Derived from Atherosclerotic Plaque
Source: Aging Dis. 2026 Oct 12;17(6):3320–36. doi: 10.14336/AD.2025.0687 (PMC13653736; doi:10.14336/AD.2025.0687)
Supplement: Supplementary file 1 [file AD-17-6-3320-s.pdf]

SUPPLEMENTARY DATA

A Signature of Senescence Based on An Analysis of Selected Markers in Primary Vascular Smooth Muscle Cells Cultured *In Vitro* and Derived from Atherosclerotic Plaque

Agata Głuchowska, Krzysztof Bojakowski, Dorota Janiszewska, Karolina Staniak, Magdalena Dudkowska, Grażyna Mosieniak

© 2025. Głuchowska A et al. Published online at http://www.aginganddisease.org/EN/10.14336/AD.2025.0687

SUPPLEMENTARY DATA

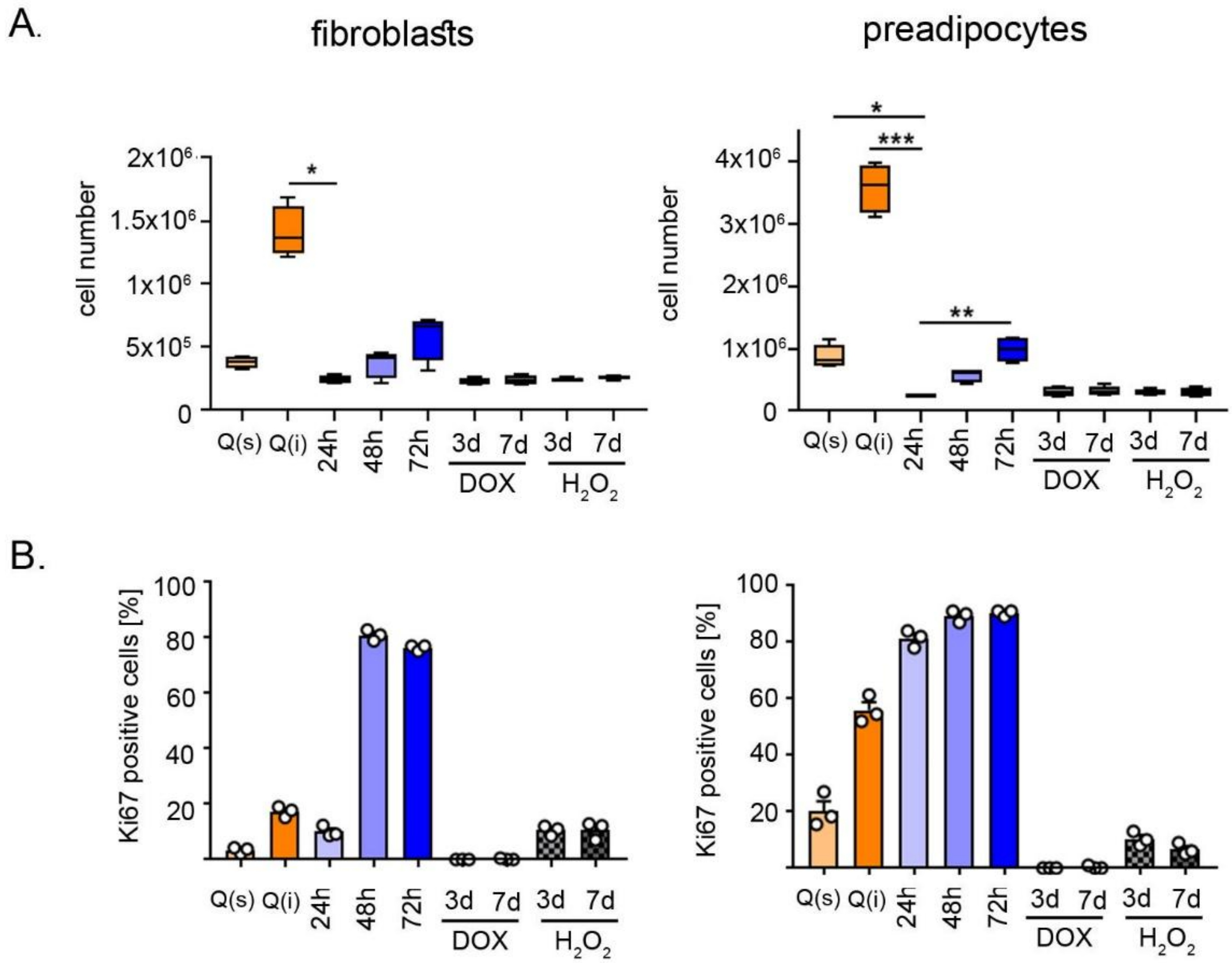

Supplementary Figure 1. Analysis of proliferation markers in fibroblasts and preadipocytes. The number (A.) and percentage of Ki67-positive cells (B.) among quiescent, proliferating and DOX- or H_2O_2-treated fibroblasts and preadipocytes were estimated. Statistical analysis was performed using Kruskal-Wallis test with FDR correction (two-stage step-up method of Benjamini, Krieger and Yekutieli) for multiple comparison; boxes: Q1, median, Q3; whiskers: min, max; *p< 0.05, **p< 0.01, ***p<0.001; N=4; bars: mean ±SEM, N=3.

© 2025. Głuchowska A et al. Published online at http://www.aginganddisease.org/EN/10.14336/AD.2025.0687

SUPPLEMENTARY DATA

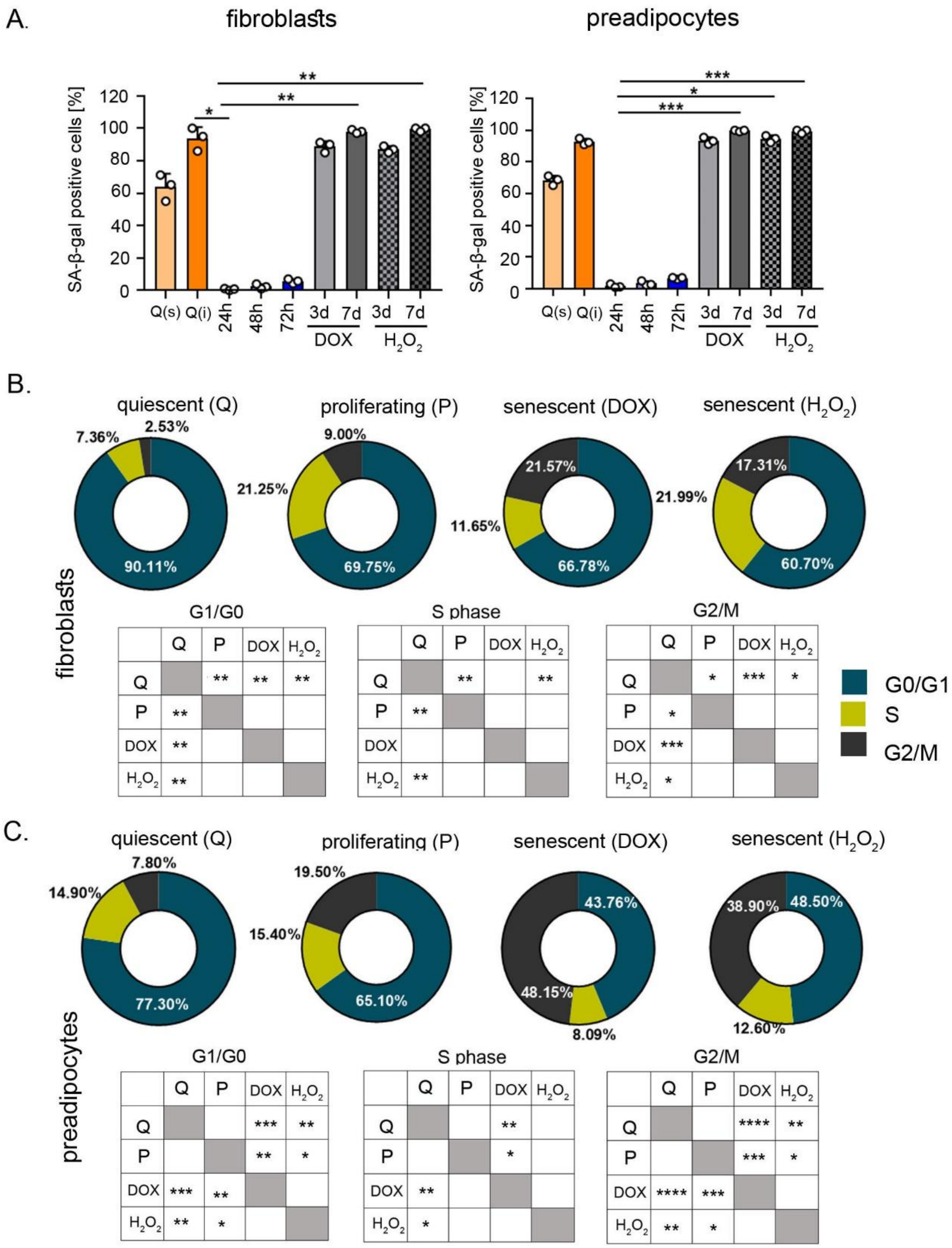

Supplementary Figure 2. Comparison of SA-β-gal activity (A.) and cell cycle progression (B., C.) among quiescent, proliferating and senescent fibroblasts and preadipocytes. The graph presents the mean ± SEM, n=3. Statistical analysis for all measurements was performed using Kruskal-Wallis test with FDR correction (two-stage step-up method of Benjamini, Krieger and Yekutieli) for multiple comparison, *p<0.05, **p< 0.01; (A.) mean ± SEM, N=3, *p<0.05, ** p< 0.01.; (B., C.) mean ±SEM, N=5, * p< 0.05, ** p< 0.01, ***p<0.001, ****p<0.0001.

© 2025. Głuchowska A et al. Published online at http://www.aginganddisease.org/EN/10.14336/AD.2025.0687

SUPPLEMENTARY DATA

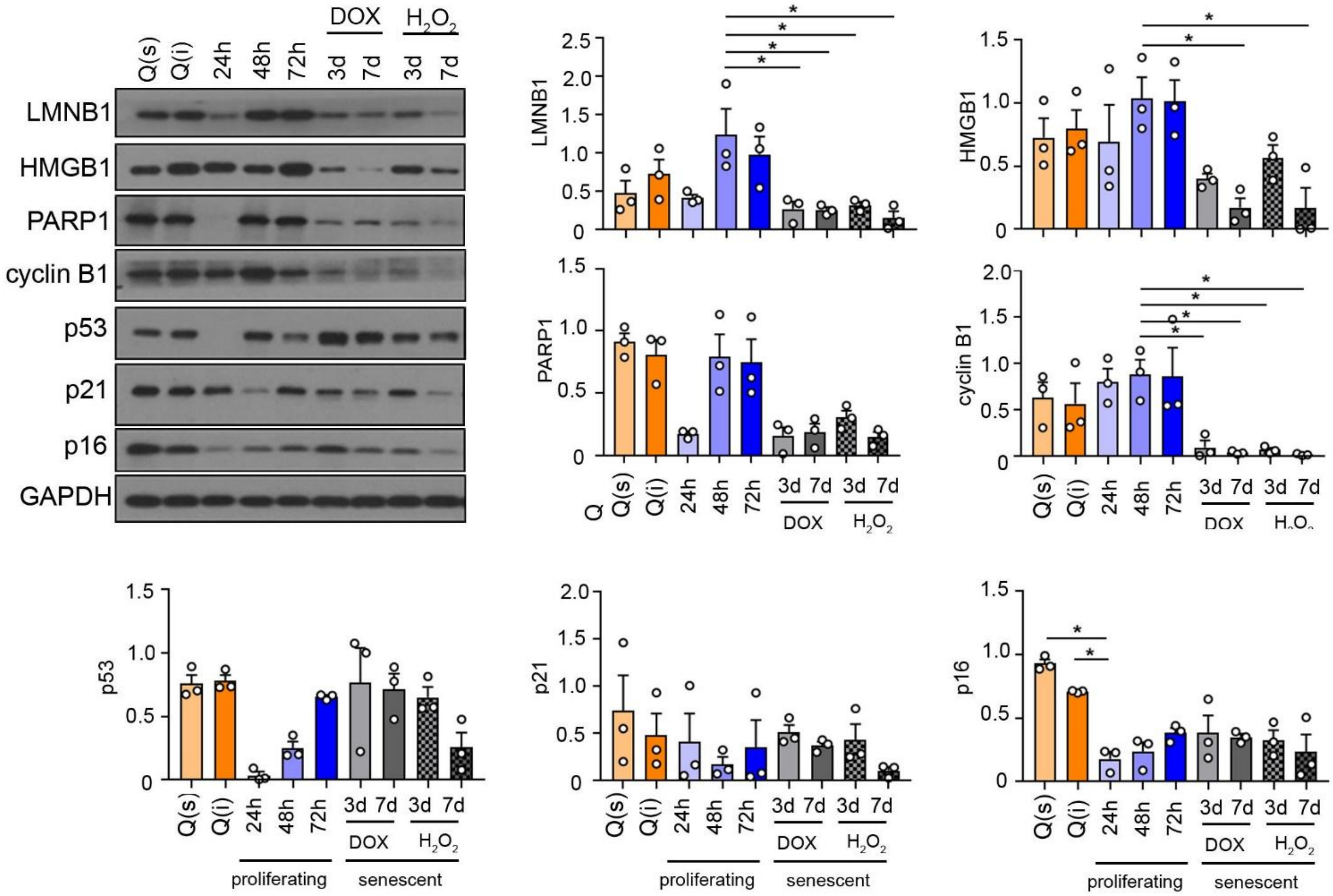

Supplementary Figure 3. Expression of selected proteins in quiescent, proliferating and senescent preadipocytes. Western blotting analysis of Lamin B1 (LMNB1), HMGB1, PARP1, cyclin B1, p53, p21 and p16 expression. The level of GAPDH served as a loading control. Representative blots are shown (left). Densitometric analysis was performed, the data for each protein were normalized to the GAPDH level, and the results in the graphs are presented as the means ±SEM, N=3. Statistical analysis was performed using Kruskal-Wallis test with FDR correction (two-stage step-up method of Benjamini, Krieger and Yekutieli) for multiple comparison, *$p<0.05$.

© 2025. Głuchowska A et al. Published online at http://www.aginganddisease.org/EN/10.14336/AD.2025.0687

SUPPLEMENTARY DATA

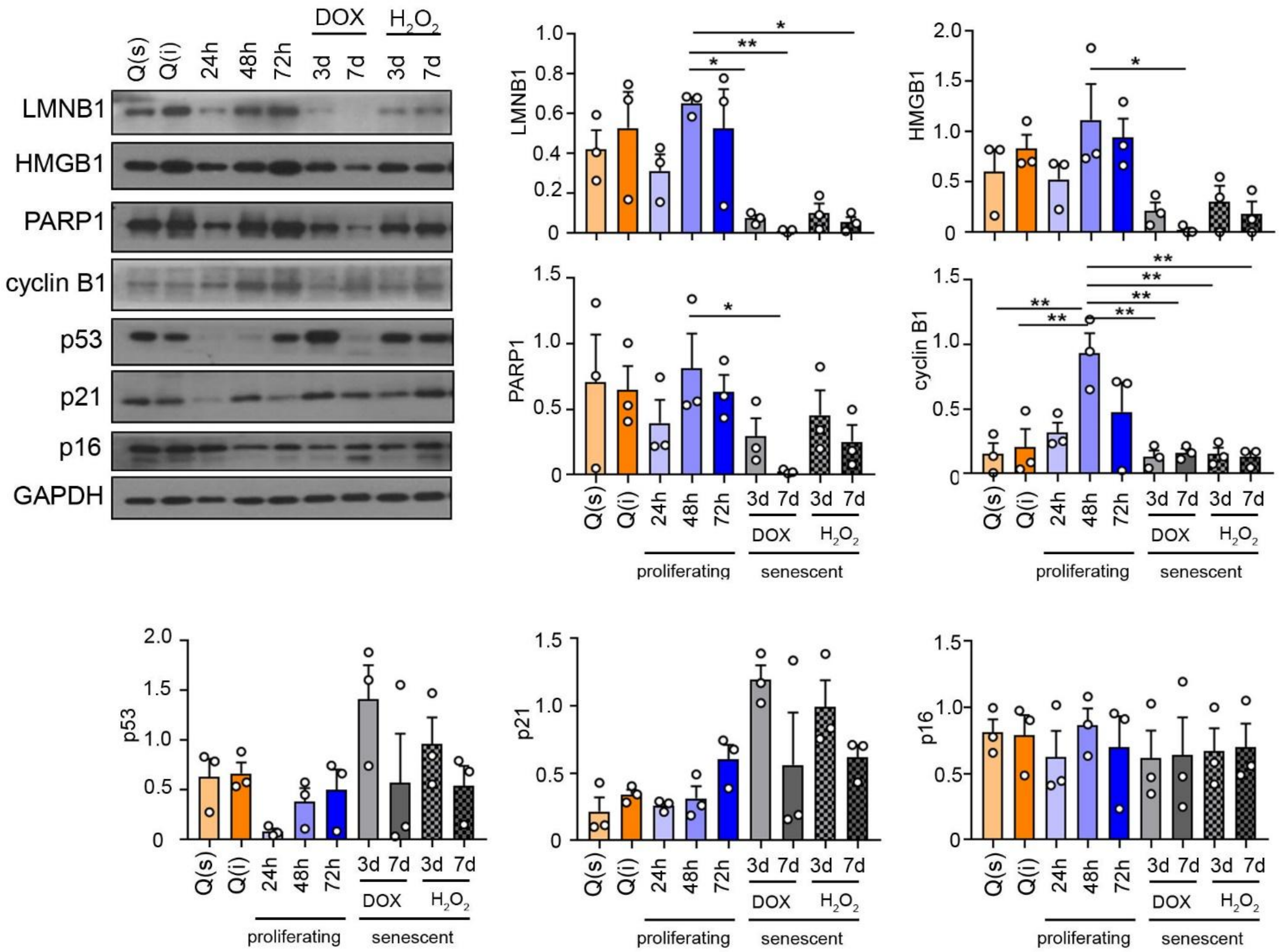

Supplementary Figure 4. **Expression of selected proteins in quiescent, proliferating and senescent fibroblasts.** Western blotting analysis of Lamin B1 (LMNB1), HMGB1, PARP1, cyclin B1, p53, p21 and p16 expression. The level of GAPDH served as a loading control. Representative blots are shown (left). Densitometric analysis was performed, the data for each protein were normalized to the GAPDH level, and the results in the graphs are presented as the means ±SEMs, N=3. Statistical analysis was performed using Kruskal-Wallis test with FDR correction (two-stage step-up method of Benjamini, Krieger and Yekutieli) for multiple comparison, *$p<0.05$, **$p< 0.01$; *$p\leq 0.05$, ** $p\leq 0.01$.

© 2025. Głuchowska A et al. Published online at http://www.aginganddisease.org/EN/10.14336/AD.2025.0687
